# Nonlinear association between red cell distribution width/albumin ratio and peripheral arterial disease in the NHANES: a cross-section study with propensity score matching analysis

**DOI:** 10.3389/fcvm.2025.1513749

**Published:** 2025-01-17

**Authors:** Jinmin Cao, Zhixiong Deng, Li Xiang, Feng Wang, Ting Yang

**Affiliations:** ^1^ Department of Dermatology, Hunan Aerospace Hospital, Changsha, China; ^2^ Department of Cardiovascular Medicine, Hunan Aerospace Hospital, Changsha, China

**Keywords:** RDW/ALB, peripheral arterial disease, red cell distribution width (RDW), NHANES, cross-sectional

## Abstract

**Background:**

Peripheral arterial disease (PAD) is a prevalent cardiovascular condition that affects up to 200 million people globally, and has significant implications for morbidity and mortality. Recent studies have identified the red cell distribution width-to-albumin ratio (RDW/ALB) as a potential systemic inflammatory marker that is correlated with several cardiovascular and inflammatory diseases including PAD. However, the comprehensive role of RDW/ALB in PAD remains underexplored. The objective of this study was to elucidate the relationship between RDW/ALB and PAD and to provide novel insights into the prevention and treatment of PAD.

**Methods:**

This retrospective cross-sectional study leveraged data from the NHANES data (1999–2004) identifying PAD using ankle-brachial index measurements (<0.90). The association with PAD was assessed using multivariable logistic regression, complemented by a restricted cubic spline for nonlinearity, and propensity score methods for robustness checks, including propensity score matching and subgroup analyses.

**Results:**

This study included 6,421 participants, 452 of whom had PAD. The mean age was 70.1 years; the patients were predominantly male. After adjustment, the RDW/ALB ratio showed a significant association with PAD (OR 1.71, 95% CI 1.29–2.26, *p* < 0.001). After quartiles of RDW/ALB, the risk of PAD was determined to be 2.03 (95% CI 1.31–3.14) in the Q4 group compared with the Q1 group in the adjusted complete model. The restricted sample cubic spline of RDW/ALB and risk of developing PAD demonstrated a nonlinear relationship. The risk of PAD increased considerably with higher RDW/ALB ratios less than 4.08. Subgroup and PSM analyses underscored the consistency of these findings.

**Conclusions:**

The study demonstrated a significant association between RDW/ALB and PAD, with a nonlinear relationship and a threshold effect. Further prospective clinical research is required to validate the relationship between the RDW/ALB ratio and PAD.

## Introduction

In 2018, approximately 200 million people worldwide were predicted to suffer from peripheral arterial disease (PAD). More than 50% of individuals with PAD are asymptomatic (1). PAD is a systemic, progressive vascular disease characterized by the accumulation of fatty deposits along the arterial wall, leading to luminal narrowing and obstructive lesions. These lesions are most frequently found in the lower extremities (2). PAD not only causes ischemic ulcers and lower limb amputations but can also greatly increase the risk of cardiovascular events and mortality (3, 4). After coronary heart disease and stroke, PAD is currently the third most common cause of morbidity from atherosclerotic cardiovascular disease (5). Patients with PAD may face severe health risks that cannot be disregarded, as well as major medical and financial obligations. Therefore, it is crucial to identify, diagnose, and treat PAD as soon as possible.

Red cell distribution width (RDW) is a part of the complete blood count and is often used as an indicator of iron deficiency anemia (6). Some clinical studies have shown that an increase in RDW may be significantly related to the risk of acute pancreatitis, chronic kidney disease, burns, cancer, diabetes, and deep venous thrombosis (7–11). In addition, erythrocyte width is considered a risk factor for increased mortality and morbidity in the general population (12). Especially in patients with cardiovascular diseases, including acute coronary syndromes, peripheral arterial disease, atrial fibrillation, heart failure, hypertension, and ischemic stroke (13–18). Serum albumin is synthesized only in the liver, and is a common biochemical marker. Albumin (ALB) is a negative acute-phase protein and its synthesis rate is negatively correlated with inflammatory activity (19). The RDW/ALB ratio is a readily available systemic marker of inflammation. According to previous studies, patients with diabetic retinopathy who have a larger red blood cell distribution width/albumin ratio are at a higher risk of dying from cardiovascular illnesses or all causes combined (19, 20). In patients with diabetic foot ulcers, RDW and the RDB/ALB ratio have been found to be independent predictive factors for all-cause mortality (21). Therefore, we aimed to explore the association between the RDW/ALB ratio and PAD in a cross-sectional study involving a US population.

## Materials and methods

### Study population

This study used data from the National Health and Nutrition Examination Survey (NHANES) cycles conducted between 1999 and 2004. NHANES is a 2-year cross-sectoral, stratified, multistage probability cluster survey designed to represent the non-institutionalized US civilian population. NHANES provides a publicly available dataset rich in demographic, socioeconomic, dietary, and health-related factors. NHANES data are publicly accessible and were obtained from the official NHANES website (http://www.cdc.gov/nchs/nhanes/). We included 6,421 of 31,126 participants by excluding those with missing data for ankle brachial blood pressure index (ABPI), red cell distribution width, albumin, and those with an ankle-brachial index (ABI) >1.4 (Supplementary Figure S1). This study was approved by the National Center for Health Statistics Research Ethics Review Board approved this study.

### Ascertainment of PAD

PAD was defined based on the ABI, a widely accepted noninvasive diagnostic measure for identifying individuals at risk of PAD. In the NHANES lower-extremity examination, systolic blood pressure was recorded in the right arm and the posterior tibial arteries of both ankles with an 8 MHz Doppler probe while each participant were supine. The ABI for each leg was calculated by dividing the average systolic blood pressure (ASBP) of the ankle by the arm ASBP. In the context of this study, an ABI of less than 0.90 in either leg indicated PAD, and participants with an ABI > 1.4 were excluded due to non-compressible arteries (22).

### Ascertainment of RDW and ALB

RDW was calculated using the complete blood count and quantified using a Beckman Coulter MAXM analyzer, as specified in the NHANES Laboratory/Medical Technologists Procedures Manual. The level of ALB, a biochemical indicator of nutritional status and a significant component of colloidal osmotic pressure, was assessed using the DcX800 technique, which employs a bichromatic digital endpoint approach. The ratio of RDW to ALB is indicated as RDW/ALB.

### Covariates

Covariates were selected with reference from previous studies, mainly including baseline characteristics, physical examination, comorbidities and laboratory tests, and medication. Baseline variables encompassed age, sex, race, education, PIR, marital status, smoking and alcohol consumption habits.

### Statistical analysis

The Free Statistics analytic platform (Version 1.9.2, Beijing, China, http://www.clinicalscientists.cn/freestatistics) and R Statistical Software (Version 4.2.2, http://www.R-project.org, The R Foundation) were used for statistical analyses. Categorical variables are given as frequencies, while continuous data are provided as mean ± standard deviation. Baseline characteristic differences were compared using independent sample *t*-tests for continuous variables and *χ*^2^ tests for categorical variables. To evaluate the association between RDW/ALB and PAD, multivariable logistic regression analysis was conducted.

Odds ratios (ORs) and 95% confidence intervals (CIs) were calculated in the analyses. From the crude unadjusted model, three models were constructed to adjust for confounding variables at different levels. Model 1 was adjusted for basic characteristics including age, sex, race, ethnicity, educational level, family income, and marital status. Model 2 included additional factors such as BMI, cardiovascular disease, stroke, family history of diabetes, hyperlipidemia, alcohol status, and smoking status. Model 3 included additional factors: HbA1c, total cholesterol, hemoglobin, MCV, and eGFR. Subgroup analyses were conducted to investigate the correlation between the RDW/ALB ratio and PAD based on age, sex, BMI, poverty income ratio (PIR), diabetes, hypertension, cardiovascular disease, HbA1c, and eGFR categories. We also performed nonlinearity and threshold analyses using a restricted cubic spline model to explore the potential non-linear relationship between RDW/ALB and PAD. Missing data for covariates were addressed using multiple imputations when the percentage of missing values exceeded 10%. Propensity score matching (PSM) was used to reduce pre-matching differences in patient characteristics to guarantee the validity of our results. Risk analysis was conducted after the PSM analysis, with several changes made to verify the consistency of the connection. Statistical significance was set at *p* < 0.05.

## Results

### Participants and baseline characteristics

The average age of the participants with PAD was 70.1 years, with a higher proportion of males than females (51.3%) (Supplementary Table S1). PAD is associated with RDW, ALB, and the RDW/ALB ratio. When stratified by RDW/ALB quartiles, the risk of PAD increased proportionally with an elevation in the RDW/ALB ratio.

The RDW/ALB ratio threshold of 2.953 was determined using a dichotomous categorization method. Before PSM, the participant group with RDW/ALB ratio less than 2.953 (*N* = 3,190) had a mean age of 57.10, contrasting with the group with RDW/ALB ratio of 2.953 or higher (*N* = 3,231), with a mean age of 61.83 years. Statistically significant intergroup disparities across a range of factors are shown as standardized mean differences (SMDs) between the groups in the table. Following PSM, each group's sample size was balanced at *N* = 2,047, and the distribution of sex (48.8% female in both groups) and age (SMD = 0.012) showed minor discrepancies, indicating a successful matching procedure. A well-matched, similar study group was indicated by the post-PSM analysis, which showed a balanced distribution across all assessed factors, including race, education level, marital status, PIR, BMI, and health-related indicators, with SMDs close to zero (Table 1).

**Table 1:** Participant characteristics by RDW/ALB ratio groups before and after propensity score matching (PSM).

Characteristics	Before propensity score		SMD	After propensity score		SMD
	<2.953	≥2.953		<2.953	≥2.953	
Sample size, *N*	3,190	3,231		2,047	2,047	
Age, mean (SD)	57.10 (11.98)	61.83 (13.20)	0.375	59.99 (12.22)	59.84 (12.70)	0.012
Sex, female. *n* (%)	1,338 (41.9)	1,774 (54.9)	0.262	999 (48.8)	999 (48.8)	<0.001
Race/ethnicity, *n* (%)			0.353			0.044
Non-Hispanic White	741 (23.2)	620 (19.2)		444 (21.7)	450 (22.0)	
Non-Hispanic Black	116 (3.6)	137 (4.2)		84 (4.1)	92 (4.5)	
Mexican American	1,916 (60.1)	1,611 (49.9)		1,177 (57.5)	1,138 (55.6)	
Other	417 (13.1)	863 (26.7)		342 (16.7)	367 (17.9)	
Education level, *n* (%)			0.162			0.02
Blow high school	928 (29.1)	1,157 (35.8)		663 (32.4)	682 (33.3)	
High school	745 (23.4)	772 (23.9)		483 (23.6)	479 (23.4)	
Above high school	1,517 (47.6)	1,302 (40.3)		901 (44.0)	886 (43.3)	
Martial, *n* (%)			0.263			0.024
Married	2,324 (72.9)	1,966 (60.8)		1,387 (67.8)	1,392 (68.0)	
Living alone	693 (21.7)	1,061 (32.8)		539 (26.3)	545 (26.6)	
Never married	173 (5.4)	204 (6.3)		121 (5.9)	110 (5.4)	
PIR, *n* (%)			0.239			0.008
Low	693 (21.7)	936 (29.0)		516 (25.2)	509 (24.9)	
Medium	1,148 (36.0)	1,280 (39.6)		779 (38.1)	785 (38.3)	
High	1,349 (42.3)	1,015 (31.4)		752 (36.7)	753 (36.8)	
BMI, kg/m^2^, mean (SD)	27.56 (4.73)	29.11 (6.11)	0.284	28.18 (4.94)	28.18 (5.45)	0.001
Diabetes, *n* (%)	437 (13.7)	619 (19.2)	0.148	335 (16.4)	331 (16.2)	0.005
Hypertensive, *n* (%)	1,524 (47.8)	1,900 (58.8)	0.222	1,093 (53.4)	1,066 (52.1)	0.026
Cardiovascular disease, *n* (%)	152 (4.8)	232 (7.2)	0.102	125 (6.1)	114 (5.6)	0.023
Stroke, *n* (%)	74 (2.3)	163 (5.0)	0.145	68 (3.3)	59 (2.9)	0.025
Diabetes family history, *n* (%)	1,532 (48.0)	1,617 (50.0)	0.04	995 (48.6)	974 (47.6)	0.021
Hyperlipidemia, *n* (%)	130 (4.1)	144 (4.5)	0.019	83 (4.1)	77 (3.8)	0.015
Smoking status, *n* (%)			0.074			0.015
Never	1,448 (45.4)	1,498 (46.4)		921 (45.0)	909 (44.4)	
Former	1,139 (35.7)	1,052 (32.6)		706 (34.5)	707 (34.5)	
Now	603 (18.9)	681 (21.1)		420 (20.5)	431 (21.1)	
eGFR, *n* (%)	3,043 (95.4)	2,817 (87.2)	0.294	1,908 (93.2)	1,940 (94.8)	0.066
Total cholesterol, mean (SD)	212.08 (40.31)	207.52 (42.62)	0.11	210.05 (38.86)	210.14 (42.25)	0.002
HbA1c (%), mean (SD)	5.63 (0.99)	5.87 (1.20)	0.222	5.74 (1.09)	5.73 (1.00)	0.006
Hemoglobin (g/L), mean (SD)	14.83 (1.24)	13.96 (1.53)	0.625	14.49 (1.18)	14.47 (1.34)	0.018
MCV, mean (SD)	91.85 (4.01)	89.88 (6.36)	0.37	91.44 (4.10)	91.33 (5.09)	0.025

Our research showed that the robustness of our observational analysis was increased by effectively minimizing pre-matching discrepancies in patient features using the four PSM approaches (Supplementary Figure S2).

### Propensity score matching and risk analysis

**Figure 1:**
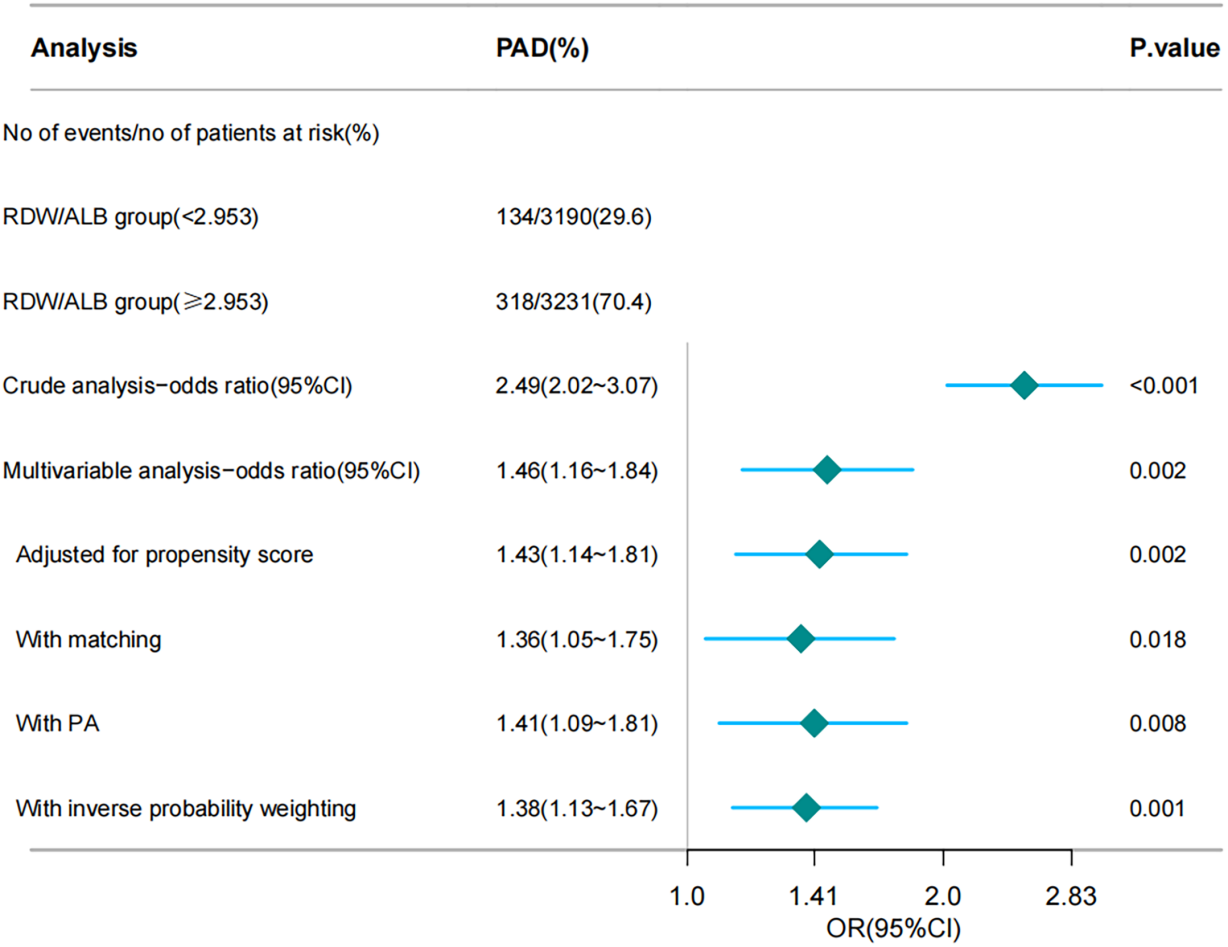
Forest plot depicting the association between the RDW/ALB ratio and the risk of peripheral arterial disease (PAD). RDW/ALB, red blood cell distribution width-to-albumin ratio; PAD, peripheral arterial disease. The chart illustrates various analytical methods, including crude analysis, multivariable analysis, propensity score adjustment, matching, PA, and inverse probability weighting, to assess the impact of the RDW/ALB ratio on the risk of patients developing PAD.

Figure 1 illustrates the ORs for PAD across the different analyses, including crude, multivariable, and various propensity score adjustments. After adjusting for multiple variables, OR decreased but remained significant (OR: 1.46; 95% CI: 1.16–1.84; *p* = 0.002). Further adjustments using PSM (OR: 1.36; 95% CI: 1.05–1.75; *p* = 0.018), propensity adjustment (OR: 1.41; 95% CI: 1.09–1.81; *p* = 0.008), and inverse probability weighting (OR: 1.38; 95% CI: 1.13–1.67; *p* = 0.001) confirmed the robustness of the association.

### Relationship between RDW/ALB and PAD

Multivariable analysis models were constructed to assess the association between RDW/ALB and the presence of PAD (Table 2). In the first adjusted model (Model 1), which accounted for sociodemographic variables, including age, sex, race, marital status, and PIR, the OR for the RDW/ALB ratio was attenuated to 1.76 (95% CI: 1.43–2.15; *p* < 0.001). Further adjustment for clinical variables in Model 2, such as BMI, hypertension, cardiovascular disease, stroke, family history of diabetes, hyperlipidemia, alcohol status, and smoking status, slightly reduced the OR to 1.66 (95% CI: 1.32–2.10; *p* < 0.001). The fully adjusted model (Model 3) included all variables from Model 2 in addition to eGFR, total cholesterol, HbA1c, hemoglobin, and MCV. The association remained statistically significant with an OR of 1.71 (95% CI: 1.29–2.26; *p* < 0.001) for the continuous RDW/ALB ratio.

**Table 2:** Association between RDW/ALB and PAD in multiple regression model.

Variable	OR (95% CI), *P* value			
	Crude	Model 1	Model 2	Model 3
RDW/ALB (continuous)	2.23 (1.88∼2.65) < 0.001	1.76 (1.43∼2.15) < 0.001	1.66 (1.32∼2.1) < 0.001	1.71 (1.29∼2.26) < 0.001
RDW/ALB (quartile)				
Q1 (2.15, 2.77)	Reference	Reference	Reference	Reference
Q2 (2.78, 2.95)	1.8 (1.26∼2.59) 0.001	1.46 (0.98∼2.17) 0.061	1.59 (1.02∼2.47) 0.003	1.53 (0.98∼2.39) 0.061
Q3 (3.96, 3.17)	2.74 (1.94∼3.85) < 0.001	1.73 (1.18∼2.53) < 0.005	1.9 (1.24∼2.91) < 0.001	1.83 (1.19∼2.82) < 0.006
Q4 (3.18, 8.37)	4.3 (3.1∼5.97) < 0.001	2.36 (1.63∼3.43) < 0.001	2.36 (1.55∼3.58)	2.03 (1.31∼3.14) 0.002
*P* for trend	<0.001	<0.001	<0.001	<0.001

Crude model: unadjusted model.

Model 1: adjusted for sociodemographic variables (Age, Sex, Race, Martial status, PIR).

Model 2: Model 1 and BMI, hypertensive, cardiovascular disease, stroke, family history of diabetes, hyperlipidemia, alcohol status, smoking status.

Model 3: adjusted for Model2, HbA1c, Total cholesterol, Hemoglobin, MCV, eGFR.

RDW, red cell distribution width; ALB, albumin; RDW/ALB, red cell distribution width/albumin ratio; BMI, Body mass index; PIR, Poverty income ratio; TC, total cholesterol; HbA1c, Glycosylated hemoglobin; HGB, hemoglobin; MCV, mean corpuscular volume; eGFR, estimated glomerular filtration rate.

When analyzed by quartiles, the highest quartile (Q4) of the RDW/ALB ratio demonstrated the strongest association with PAD in all models. The OR for Q4 was 4.3 (95% CI: 3.1–5.97; *p* < 0.001) in the crude model and reduced to 2.03 (95% CI: 1.31–3.14; *p* = 0.002) in the fully adjusted Model 3. A significant trend across quartiles was observed in all models (*p* < 0.001).

### Nonlinearity and threshold analysis

**Figure 2:**
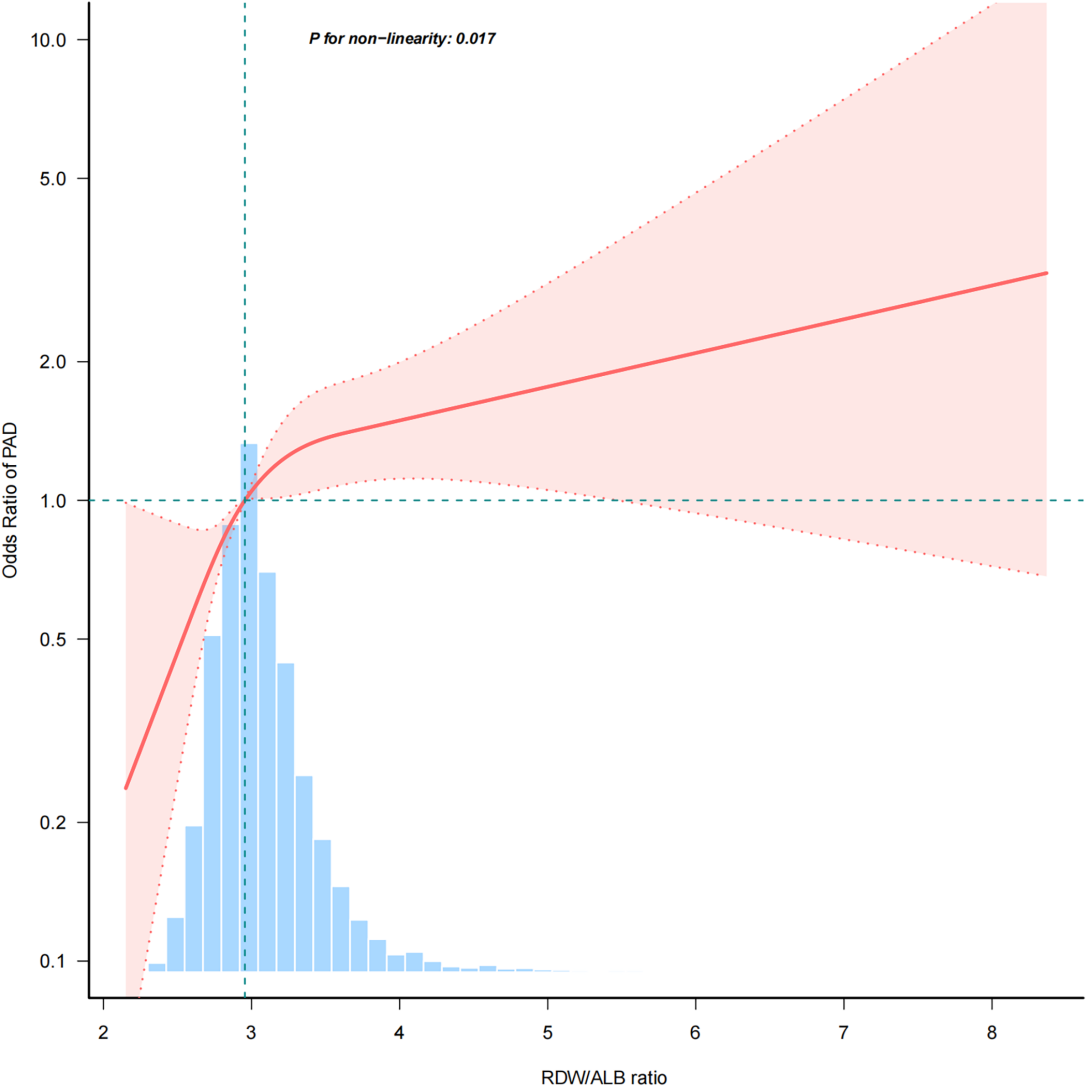
A nonlinear relationship between RDW/ALB and risk of PAD. RDW/ALB, red blood cell distribution width to albumin ratio; PAD, peripheral arterial disease.

The association between the RDW/ALB ratio and PAD was nonlinear (*p* = 0.017) in the restricted cubic spline model (Figure 2). In the two-piecewise regression models, the adjusted OR for developing PAD was 1.988 (95% CI: 1.378–2.867; *p* < 0.001) with RDW/ALB < 4.08. This suggests that for every unit increase in the RDW/ALB ratio below 4.08, there was a nearly two-fold increase in the odds of PAD. Conversely, there was no association between the RDW/ALB ratio and peripheral arterial disease in participants with RDW/ALB ≥ 4.08 (Table 3).

**Table 3:** Inflection point analysis of RDW/ALB and PAD.

RDW/ALB ratio	Adjust OR (95% CI)	*P* value
<4.08	1.988 (1.378∼2.867)	<0.001
≥4.08	0.323 (0.024∼4.304)	0.393
Likelihood ratio test	–	0.025

Adjusted for age, sex, race, marital status, PIR, BMI, hypertensive, cardiovascular disease, stroke, family history of diabetes, hyperlipidemia, alcohol status, smoking status, HbA1c, total cholesterol, hemoglobin, MCV, eGFR.

### Subgroup analyses of factors influencing the association between RDW/ALB and presence of PAD

Subgroup analysis revealed that the correlation between RDW/ALB and PAD was not significantly different among the subgroups. The study indicated a consistent relationship that was unaffected by race, education, marital status, PIR, diabetes, hypertension, cardiovascular disease, diabetes family history, hyperlipidemia, smoking status, eGFR, cholesterol, HbA1c, hemoglobin, and MCV (Supplementary Figures S3, S4).

## Discussion

In this retrospective study, a high RDW/ALB ratio was found to be associated with an increased risk of developing PAD in 452 adult US outpatients with PAD. Patients with PAD exhibited a higher RDW/ALB ratio than those without PAD. Even after adjusting for potential confounding variables, the risk of developing PAD remained significant with a high RDW/ALB ratio. The results of subgroup analyses remained robust after stratification for relevant confounders. According to the findings of this study, RDW/ALB is an independent risk factor for the development of PAD.

In this study, PAD was found to be associated with increased RDW/ALB, which is consistent with the patterns observed in other cardiovascular diseases. Moreover, a high RDW/ALB ratio is a strong predictor of several unfavorable outcomes related to cardiovascular disease such as mortality after percutaneous coronary interventions, early death from ischemic stroke, complications in patients with sepsis experiencing atrial fibrillation, and the presence of carotid plaques in patients with coronary heart disease at different metabolic stages (23–26).

PAD progression involves a complex interplay of factors such as atherosclerotic obstructions, inflammation, oxidative stress, endothelial dysfunction, and impaired vasodilation (27). Atherosclerotic plaques in peripheral arteries have more fibrotic components, fewer lipids, and fewer inflammatory cells, making them more stable and less likely to rupture, and plaque occlusion leads to narrowed and reduced blood flow to the extremities (28). Studies suggest that cytokines, matrix metalloproteinases, selectins, intracellular adhesion molecules, vascular cell adhesion molecules, C-reactive protein, and fibrinogen are involved in the initiation and progression of atherosclerosis, which influences the onset and development of PAD (29). PAD is closely linked to oxidative stress, with studies highlighting the role of reactive oxygen species (ROS) and redox signaling in disease progression (30, 31). Oxidative stress in PAD is characterized by increased ROS production, leading to endothelial dysfunction, arterial stiffness, and inflammation, all of which contribute to functional impairment in patients (32). By measuring 8-hydroxy-2-deoxy-2-deoxyguanosine and nitrite/nitrate, which are biomarkers of oxidative stress levels in patients with PAD, the results showed elevated markers of oxidative stress and decreased nitric oxide bioavailability in PAD (33). Endothelial dysfunction is associated with increased oxidative stress and decreased nitric oxide bioavailability, leading to endothelial damage and dysfunction. It not only leads to an imbalance of vasoactive substances (e.g., nitric oxide and endothelin-1) but also results in impaired vasodilation, increased inflammation, and enhanced thrombosis, all of which are key factors in the development and progression of PAD (31, 34, 35). In addition, endothelial dysfunction can lead to an increase in endothelial-medial or plaque thickness, which, in turn, affects peripheral vascular blood flow (36).

An increased RDW is associated with impaired erythropoiesis and shortened erythrocyte lifespan, which are commonly observed in iron deficiency anemia, hemolytic anemia, and nutritional deficiencies (including iron, vitamin B12, and folate deficiencies) (37, 38). Inflammation is an important factor in the progression of PAD. Inflammation leads to dysfunctional bone marrow, inhibiting the normal production of red blood cells and causing the release of immature red blood cells into the circulation, consequently increasing RDW levels (39, 40). Moreover, chronic inflammation accelerates erythrocyte destruction, leading to further changes in erythrocyte size (41). Additionally, *in vitro* studies have demonstrated that oxidative stress can affect erythrocyte size, leading to increased RDW, emphasizing the role of oxidative stress in influencing RDW. There has been no evidence of endothelial cell damage causing RDW elevation (42). Elevated RDW is associated with reduced erythrocyte deformability, thereby impairing microcirculation and increasing cardiovascular disease risk, with cell morphology and inflammation being influential factors (43, 44).

This study, which used data from the NHANES (1999–2004) database, has several limitations that warrant consideration. First, the cross-sectional design precluded the establishment of causality between the RDW/ALB ratio and PAD, limiting the ability to infer temporal relationships or assess changes over time. Second, the reliance on ABI measurements to identify PAD might miss subclinical or early stage cases, potentially leading to an underestimation of disease prevalence. Third, the dataset lacked detailed information on potential confounders, such as inflammatory biomarkers, medication use, and lifestyle factors, which could influence the RDW/ALB ratio and PAD risk. Fourth, the retrospective nature of the study and the reliance on self-reported data may have introduced recall bias and inaccuracies in the participant-reported medical histories. Fifth, although PSM and other statistical adjustments were used to mitigate confounding factors, residual confounding factors cannot be entirely ruled out. Although we have adjusted for the selected confounding variables used in the article, there may still be unmeasured confounders that affect the results of the study. We calculated an E-value to assess the sensitivity to unmeasured confounding. RDW/ALB was associated with the prevalence of PAD in peripheral arterial disease with an OR = 1.71 (95% CI, 1.29–2.26) and an *E*-value = 2.81 (Supplementary Figure S5). This means that if all unmeasured covariates were associated with the relative risk of PAD, the residual confounding would have to be greater than 2.81 to affect the observed association.

## Conclusion

Peripheral artery disease has been found to be independently correlated with the RDW/ALB ratio. Further prospective research is needed to validate these results and examine the usefulness of the RDW/ALB ratio in PAD prediction modeling.

## Data Availability

Publicly available datasets were analyzed in this study. This data can be found here: https://www.cdc.gov/nchs/nhanes/index.html.
