# Supplementary material for: Nonlinear association between red cell distribution width/albumin ratio and peripheral arterial disease in the NHANES: a cross-section study with propensity score matching analysis
Source: Front Cardiovasc Med. 2025 Jan 17;12:1513749. doi: 10.3389/fcvm.2025.1513749 (PMC11782218; doi:10.3389/fcvm.2025.1513749)
Supplement: Supplementary file 1 [file Datasheet1.docx]

**Supplementary material:**


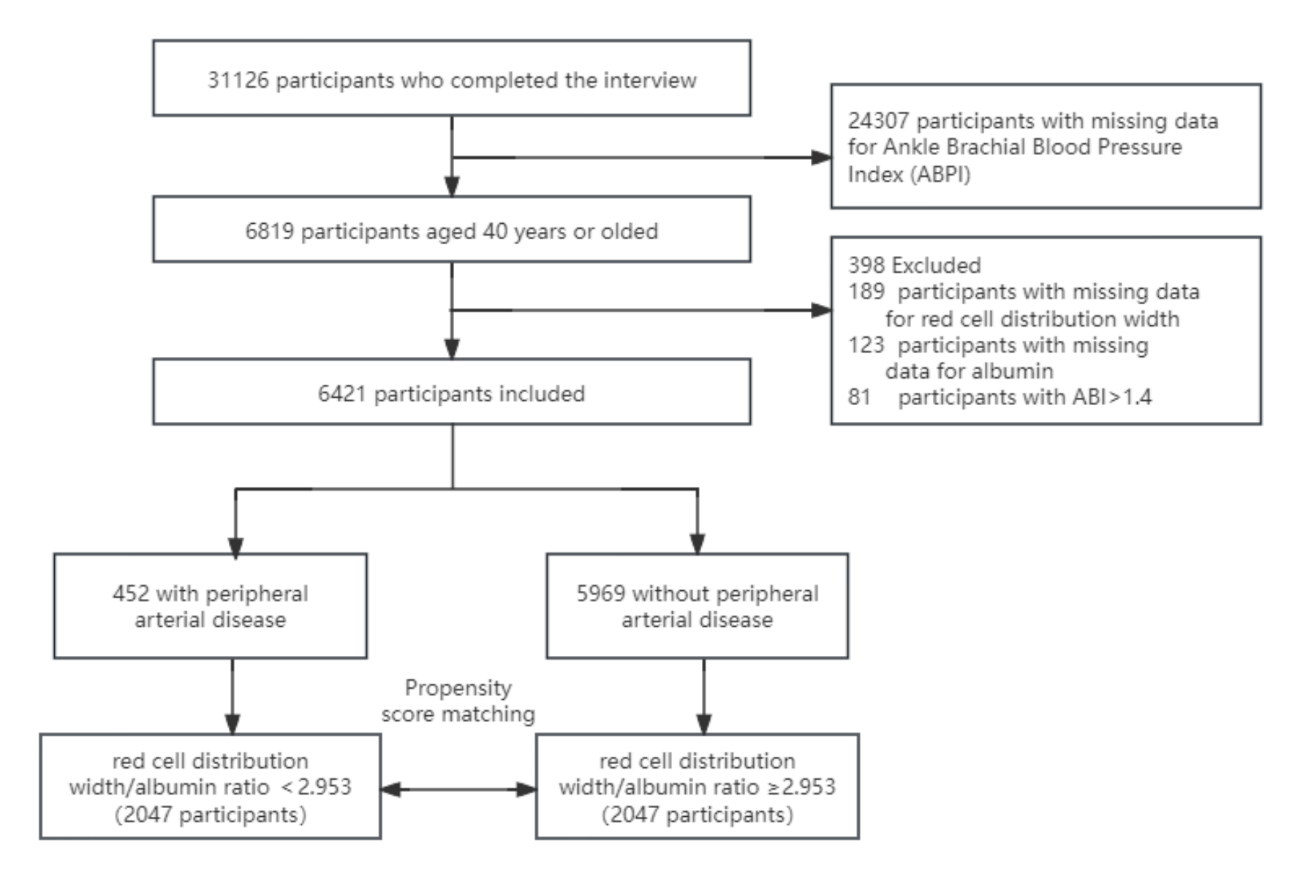


**Figure S1. The flow chart of the study.**

**Table S1 General characteristics of the study population according to peripheral arterial disease (PAD).**

| Variables | Participants | | | |
| --- | --- | --- | --- | --- |
|  | Total | No-PAD | PAD | p |
|  | n = 6421 | n = 5969 | n = 452 |  |
| RDW, (%) | 12.8 ± 1.2 | 12.8 ± 1.1 | 13.3 ± 1.5 | < 0.001 |
| ALB, g/dL | 4.3 ± 0.3 | 4.3 ± 0.3 | 4.2 ± 0.3 | < 0.001 |
| RDW/ALB, Mean ± SD | 3.0 ± 0.4 | 3.0 ± 0.4 | 3.2 ± 0.5 | < 0.001 |
| RDW/ALB.4, n (%) |  |  |  | < 0.001 |
| Q1(2.15,2.77) | 1555 (24.2) | 1508 (25.3) | 47 (10.4) |  |
| Q2(2.78,2.95) | 1635 (25.5) | 1548 (25.9) | 87 (19.2) |  |
| Q3(3.96,3.17) | 1616 (25.2) | 1489 (24.9) | 127 (28.1) |  |
| Q4(3.18,8.37) | 1615 (25.2) | 1424 (23.9) | 191 (42.3) |  |
| Age, years | 59.5 ± 12.8 | 58.7 ± 12.5 | 70.1 ± 11.8 | < 0.001 |
| Sex, n (%) |  |  |  | 0.927 |
| Male | 3309 (51.5) | 3077 (51.5) | 232 (51.3) |  |
| Female | 3112 (48.5) | 2892 (48.5) | 220 (48.7) |  |
| Race/ethnicity, n (%) |  |  |  | 0.004 |
| Non-Hispanic White | 1361 (21.2) | 1288 (21.6) | 73 (16.2) |  |
| Non-Hispanic Black | 253 ( 3.9) | 241 (4) | 12 (2.7) |  |
| Mexican American | 3527 (54.9) | 3271 (54.8) | 256 (56.6) |  |
| Other | 1280 (19.9) | 1169 (19.6) | 111 (24.6) |  |
| Education level, n(%) |  |  |  | < 0.001 |
| blow high school | 2085 (32.5) | 1891 (31.7) | 194 (42.9) |  |
| high school | 1517 (23.6) | 1403 (23.5) | 114 (25.2) |  |
| above high school | 2819 (43.9) | 2675 (44.8) | 144 (31.9) |  |
| Marital, n(%) |  |  |  | < 0.001 |
| Married | 4290 (66.8) | 4042 (67.7) | 248 (54.9) |  |
| Living alone | 1754 (27.3) | 1568 (26.3) | 186 (41.2) |  |
| Never married | 377 ( 5.9) | 359 (6) | 18 (4) |  |
| PIR, n (%) |  |  |  | < 0.001 |
| Low | 1629 (25.4) | 1466 (24.6) | 163 (36.1) |  |
| Medium | 2428 (37.8) | 2233 (37.4) | 195 (43.1) |  |
| High | 2364 (36.8) | 2270 (38) | 94 (20.8) |  |
| BMI, kg/m^2^ | 28.3 ± 5.5 | 28.4 ± 5.5 | 28.0 ± 5.9 | 0.130 |
| Diabetes, n (%) | 1056 (16.4) | 926 (15.5) | 130 (28.8) | < 0.001 |
| Hypertensive, n (%) | 3424 (53.3) | 3075 (51.5) | 349 (77.2) | < 0.001 |
| Cardio vascular disease, n(%) | 384 ( 6.0) | 321 (5.4) | 63 (13.9) | < 0.001 |
| Stoke, n (%) | 237 ( 3.7) | 191 (3.2) | 46 (10.2) | < 0.001 |
| Family history of diabetes, n (%) | 3149 (49.0) | 2935 (49.2) | 214 (47.3) | 0.454 |
| Hyperlipidemia, n(%) | 274 ( 4.3) | 253 (4.2) | 21 (4.6) | 0.679 |
| Smoking status, n(%) |  |  |  | < 0.001 |
| Never | 2946 (45.9) | 2806 (47) | 140 (31) |  |
| Former | 2191 (34.1) | 1995 (33.4) | 196 (43.4) |  |
| Now | 1284 (20.0) | 1168 (19.6) | 116 (25.7) |  |
| eGFR, ＜60 | 561 ( 8.7) | 444 (7.4) | 117 (25.9) | < 0.001 |
| Total cholesterol, mg/dL | 209.8 ± 41.6 | 210.0 ± 41.2 | 206.8 ± 46.0 | 0.116 |
| HbA1c (%) | 5.7 ± 1.1 | 5.7 ± 1.1 | 6.0 ± 1.2 | < 0.001 |
| Hemoglobin, g/L | 14.4 ± 1.5 | 14.4 ± 1.5 | 14.1 ± 1.6 | < 0.001 |
| MCV, (%) | 90.9 ± 5.4 | 90.8 ± 5.4 | 91.4 ± 5.9 | 0.043 |

Data presented as mean ± SD or median (25, 75 percentile) or n (%).

Differences were analyzed by ANOVA, Kruskal–Wallis, and chi-squared test between vanin-1 tertiles as appropriate. An alpha-level of p < 0.05 (two-tailed) was considered statistically significant.

PAD, peripheral arterial disease; RDW, red cell distribution width; ALB, albumin; RDW/ALB, red cell distribution width/albumin ratio; BMI, Body mass index; PIR, Poverty income ratio; TC, total cholesterol; HbA1c, Glycosylated hemoglobin; HGB, hemoglobin; MCV: mean corpuscular volume; eGFR: estimated glomerular filtration rate;


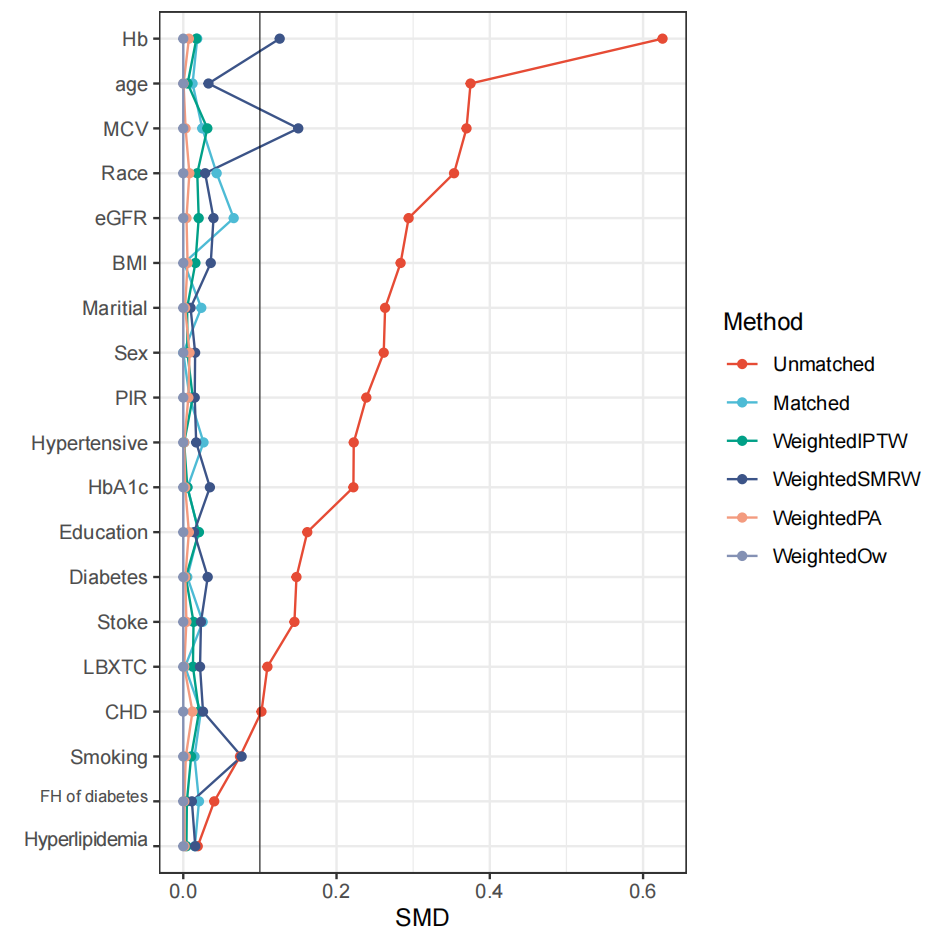


**Figure S2 Standardized Mean Differences (SMD) for Baseline Characteristics Across Different Matching and Weighting Methods.**

The methods compared include unmatched, matched, inverse probability of treatment weighting (IPTW), stabilized inverse probability of treatment weighting (SMRW), propensity score weighting (PA), and overlap weighting (Ow). The SMDs are displayed on a scale from 0.0 to 0.2 with a lower SMD indicating better balance between treatment group.


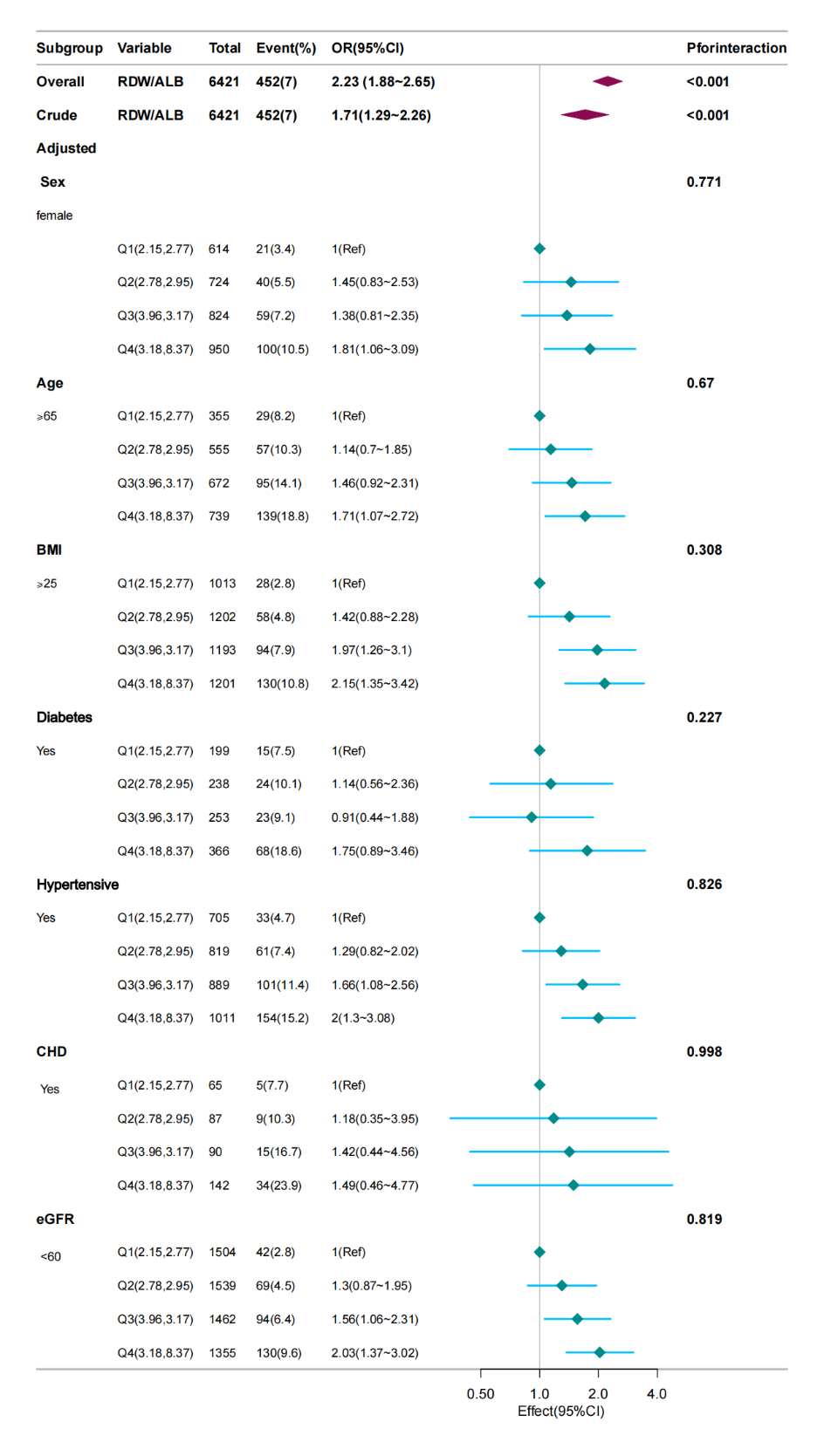


**Figure S3 Subgroup analyses and Forest plot**

**
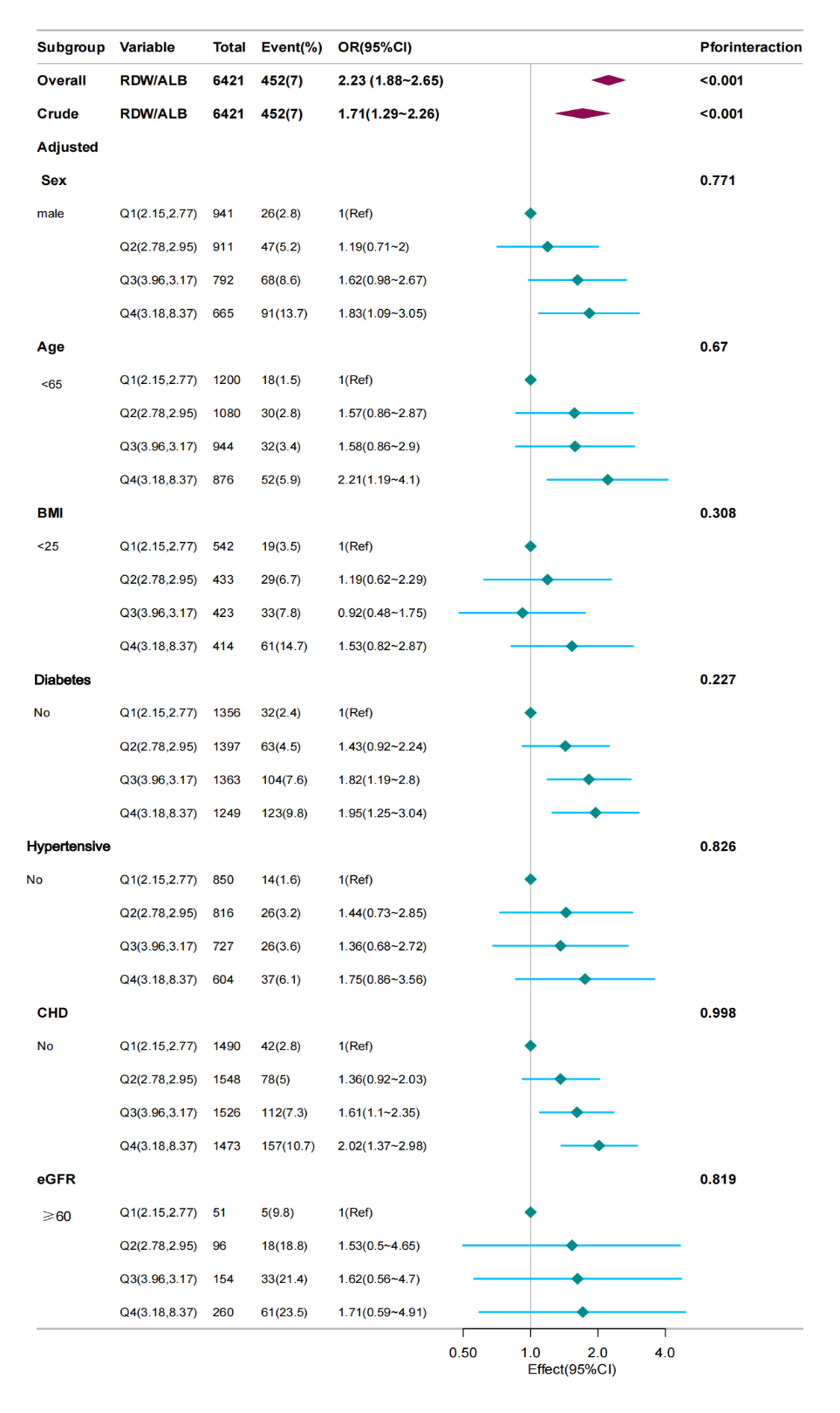
**

**Figure S4 Subgroup analyses and Forest plot.**


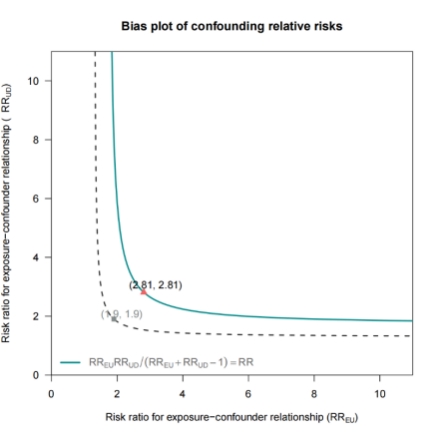


**Figure S5 Bias plot of confounding relative risks**
